# Subclinical atherosclerosis burden in non-diabetic hypertensives treated in primary care center: the IMTABI study

**DOI:** 10.1186/s12875-023-01997-8

**Published:** 2023-02-09

**Authors:** José M. Ramírez-Torres, Antonio López-Téllez, María J. Ariza, José Rioja, Natalia García-Casares, Elena E. González Rodríguez, José A. Ramírez García, Miguel A. Sánchez Chaparro, Miguel A. Barbancho, Pedro Valdivielso

**Affiliations:** 1 Centro de Salud Puerta Blanca, Servicio Andaluz de Salud, Málaga, Spain; 2 Lipids and Atherosclerosis Laboratory, Department of Medicine and Dermatology, Centro de Investigaciones Medico Sanitarias (CIMES), Instituto de Investigación Biomédica de Málaga (IBIMA), University of Málaga, Málaga, Spain; 3 Servicio de Medicina Interna, Hospital Universitario Virgen de La Victoria, Málaga, Spain; 4 Departamento de Fisiología Humana, Universidad de Málaga, Málaga, Spain

**Keywords:** Hypertension, Cardiovascular risk assessment, Carotid ultrasonography, Atherosclerotic burden, Ankle-brachial index

## Abstract

**Background:**

Identifying patients at high risk of cardiovascular disease in primary prevention is a challenging task. This study aimed at detecting subclinical atherosclerosis burden in non-diabetic hypertensive patients in a primary care centre.

**Methods:**

Clinical, anthropometric and analytical data were collected from patients with hypertension who were free from clinical vascular disease and diabetes. The cardiovascular risk was assessed using the SCORE system. Subclinical atherosclerosis burden was assessed by carotid ultrasonography (intima-medial thickness [IMT] and plaque) and measurement of the ankle-brachial index (ABI).

**Results:**

Out of 140 patients, 59 (42%) have carotid plaque, 32 (23%) have IMT higher than 75% and 12 (9%) have an ABI < 0.9. Total atherosclerosis burden was present in 91 (65%) of the subjects. Consequently, 59 (42%) patients were re-classified into the very high-risk category. In multivariate analyses, smoking, creatinine levels and duration of hypertension were associated with atherosclerosis burden. In contrast, only smoking and age were associated with the presence of carotid plaque. Almost 90% of patients were treated with hypotensive drugs, half of them combined several drugs and 60% were well-controlled. Only 30% received statins in monotherapy and only less than 20% had an LDL cholesterol < 100 mg/dL.

**Conclusions:**

In non-diabetic hypertensive patients managed at a primary care centre, 4 out of 10 had subclinical atherosclerosis burden and were re-classified into the very high- risk category. There was clear undertreatment with lipid-lowering drugs of most LDL cholesterol inappropriate levels, according to current clinical guidelines.

## Introduction

The traditional strategy based on assessing risk factors is not very sensitive for identifying high-risk or very high-risk patients in primary prevention [1]. Therefore, clinical practice guidelines recommend the use of non-invasive tests to detect the presence of subclinical atherosclerosis and improve the prediction of vascular risk (VR) in patients [2].

Imaging tests detect organic damage in asymptomatic individuals [3]. Subclinical damage is an independent determinant of VR and allows for a better re-classification of patients, especially those with a moderate VR, according to the SCORE risk tables. A clear dissociation was observed between the VR stratification by traditional vascular risk factors (VRF) and the presence of atheromatous plaque, as 25% of individuals with low-intermediate VR present carotid atheromatosis [4].

Atherosclerotic burden (ATB) is a powerful predictor of cardiovascular events and death [5, 6] and is related to a poor prognosis after suffering any cardiovascular event [7]. Measurement of the ankle-brachial index (ABI) and vascular ultrasound are two techniques that enable determination of the ATB and are available in primary care (PC). A low ABI (< 0.90) indicates a diagnosis of peripheral arterial disease (PAD) and advanced atherosclerosis [8, 9]. In addition, it is linked to a higher incidence of angina, myocardial infarction, congestive heart failure, the need for coronary artery bypass surgery, stroke, and carotid and peripheral vascular surgery [10, 11]. A recent meta-analysis shows that the ABI adds predictive power to VR scales based on traditional risk factors [12].

Although the US Preventive Services Task Force recommends against screening for asymptomatic carotid disease in the general population due to the high surgical risk it entails [13], carotid ultrasound is widely used to assess vascular risk in patients in primary prevention. Carotid intima-media thickness (IMT) has been considered a marker of generalized atherosclerotic disease [14] and is associated with stroke and coronary artery disease [15, 16]. An IMT > 0.9 mm is considered to be pathological [17], although it is a continuous variable with an upper limit of normality that varies depending on age. Hence, other guidelines classify as pathological an IMT above 75% based on the age, ethnicity and sex of the population [18]. An increased IMT, as an indication of subclinical disease, may reflect the consequences of a previous exposure to VRF [19].

Carotid plaques present a high predictive value for both stroke and myocardial infarction, regardless of traditional VRFs [16] and provide a higher prognostic value for myocardial infarction than IMT [20]. The presence of carotid or femoral plaques, as well as a high coronary artery calcium (CAC) score, automatically entails re-classifying the patient from a moderate to a higher risk category. In fact, the recording of significant carotid plaque allows for the patient to be classified as very high risk [2].

Triglycerides and especially postprandial particles estimated by a concentration of apolipoprotein (APO) B48 have shown a relationship between carotid atherosclerosis and peripheral arterial disease [21, 22]. APOE is known for its influence in cardiovascular disease (CVD). The ε4 isoform is associated with an increased risk of developing myocardial infarction, coronary heart disease and stroke [23]. Some research studies have also shown an association between some variants of the hepatic lipase gene (LIPC) promoter and the presence of PAD and carotid atherosclerosis [24, 25]. Because diabetes is associated with early atherosclerosis, occult PAD and hypertriglyceridemia, we decide to exclude them to know the role in subclinical disease of subjects with isolated hypertension.

The aim of this study was to assess the detection of subclinical ATB in hypertensive patients with moderate or high VR by using carotid ultrasound and ABI measurement, identify patients who need to be re-classified, and finally examine the studied variables to determine which of these are associated with ATB.

## Patients and methods

### Demographic and clinical data

A descriptive cross-sectional study was carried out at the Puerta Blanca Healthcare Centre in Malaga, Spain. The sample consisted of 140 consecutive patients who fulfilled the inclusion criteria and signed the informed consent, since July 2016 and April 2018. Inclusion criteria for the sample were: hypertensive patients of both sexes with a moderate or high vascular risk (1 to 10% score) who consecutively attended the center’s vascular risk clinic. Exclusion criteria for the sample were: patients previously diagnosed with cerebrovascular disease (ischemic stroke, cerebral hemorrhage, transient ischemic attack), heart disease (myocardial infarction, angina, coronary revascularization, heart failure), symptomatic peripheral arterial disease (intermittent claudication vascular, previous revascularization surgery), diabetes, primary hyperlipidemia, severe chronic kidney disease (estimated GFR < 30 ml/min) and secondary hypertension.

The study recorded sociodemographic variables (age, sex), anthropometric variables (body mass index (BMI), abdominal circumference) and clinical variables, namely: smoking, exercise (using the Physical Activity Guidelines for Americans questionnaire), dyslipidemia on previous lipid-lowering treatment, time evolution of hypertension, left ventricular hypertrophy observed by electrocardiogram according to Sokolow–Lyon index >3.5 mV, RaVL >1.1 mV; Cornell voltage duration product >244 mV/ms, chronic kidney disease (eGFR < 60 ml/min), systolic blood pressure (SBP), diastolic blood pressure (DBP) and blood pressure (BP) control in the last 6 months. BP was determined using a semi-automatic OMRON HEM-7154-E device; the measurement was performed on 3 times with a 5-min difference between each measurement, using the last two measurements as an average value. Hypertension is defined as values ≥140 mmHg SBP and/or ≥90 mmHg DBP [26]. Dyslipidemia was considered if total cholesterol > 200 mg/dL, trglycerides > 150 mg/dL or HDL cholesterol < 40 mg/dL (men) or < 50 mg/dL (women). Patients were only considered to be smokers if they had smoked regularly in the past 6 months, regardless of the amount. The criteria used for metabolic syndrome and abdominal obesity were those of the Adult Treatment Panel (ATP-III) [27].

### Analytical data

After a night of fasting, blood samples were taken and analytical variables were collected, namely: hematimetry, blood glucose, creatinine, glycohemoglobin A1c, total cholesterol (TC), low-density lipoprotein cholesterol (LDL-C), high-density lipoprotein cholesterol (HDL-C), triglycerides (TG), uric acid, thyroid stimulating hormone (TSH) and microalbuminuria. Variables were collected through a clinical autoanalyser, the Siemens Dimension Vista System Flex analysis system, at the clinical laboratory of the Virgen de la Victoria University Hospital in Malaga, Spain. Patients were considered to suffer from atherogenic dyslipidemia when presenting TG values ≥ 150 mg/dl and low HDL-C (< 40 mg/dl in men and < 50 mg/dl in women) [28]. The glomerular filtration rate (eGFR) was estimated using the CKD-EPI formula [29].

Apolipoprotein A1, Apolipoprotein B, ferritin and PCRus were analyzed using the Spinreact kit (Barcelona, Spain) on a Mindray BS-380 autoanalyser. Apolipoprotein B48 was measured by ELISA (Shibayagi). *APOE* genotypes and *LIPC* gene variants were analyzed through TaqMan assays with linear fluorogenic probes [30, 31]. Analyses were conducted at the Lipids and Arteriosclerosis Laboratory in the Medical and Health Research Centre (CIMES) (Centro de Investigaciones Médico Sanitarias in Spanish) at the University of Malaga (UMA).

### Vascular risk and subclinical atherosclerosis assessment

Patients’ VR was estimated using the European SCORE for low-risk countries [32]. In patients undergoing drug treatment, the total cholesterol and SBP values considered were those prior to starting their treatment.

The ABI was measured using a portable Doppler (Huntleigh with 8 MHz probe) [33]. The carotid ultrasound was performed with a SonoSite NanoMaxx ultrasound system in B-mode with a 10–5 MHz broadband linear probe. We took the lowest value obtained in both legs, at tibialis posteriores or pedia arteries. Both carotids were explored to search for plaques in the common carotid, carotid bulb, and internal and external carotids. Plaques were defined as a focal thickening > 1.5 mm or a thickening of over 50% of the surrounding IMT value [34]. The mean IMT value was obtained in the distal segment of the common carotid (1 cm before bifurcation), using automatic software (SonoSite SonoCalc IMT software, SonoSite Inc., Bothell, WA, USA) as the mean thickness average value measured in each of the 6 segments (from 3 different angles on the right and left sides). All carotid ultrasonography studies were performed by the same technician.

Patients were classified according to the presence or absence of ATB, considering ATB as less than 0.9, mean IMT > 75% of percentile for their age and sex, or considering the presence of atheromatous plaque.

### Statistical analysis

Quantitative variables are shown as mean and standard or median deviation (interquartile range) and the qualitative variables are shown as figures (%). A descriptive, bivariate (χ2, Student's t, ANOVA) and multivariate statistical analysis was performed using forward stepwise binary logistic regression (Wald), taking the ATB as the dependent variable and covariates who were associated in univariate analyses and those who have clinical relevance according to investigators (SPSS 26.0, IBM).

## Results

The study included 140 hypertensive patients aged 62 ± 9, out of which 76 (54%) were men. Among all participants, they were in moderate (40.7%) and high (59.3%) vascular risk. After conducting a carotid ultrasound and an ABI measurement, the most common damage was carotid plaque in 59 patients (42%), followed by a mean IMT > 75% in 32 (23%) and 12 (8.6%) patients with an ABI < 0.9. Considering that 10 patients with ABI < 0.9 also had plaque in the carotid and a further 2 showed a mean IMT > 75%, the total number of patients with subclinical atherosclerotic burden was 91 (65%).

**Fig. 1:**
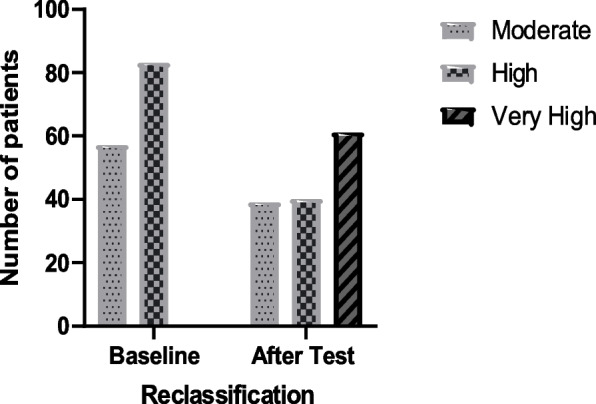
Changes in SCORE risk categories before and after vascular ultrasound and the ankle-brachial index

Out of 57 patients with a previous moderate VR, 14 (25%) had at least one carotid plaque and 1 had ABI < 0.9 (2%). Out of the 83 patients with a high VR, 45 (53%) had plaque and 11 (13%) had ABI < 0.9. As a result, 59 patients (42%) from the studied sample were re-classified as having a very high VR (Table 1 and Fig. 1).

**Table 1:** Patients affected by subclinical arteriosclerosis according to CV Risk at baseline

	Cardiovascular Risk		
	Moderate	High	*p*
IMT > p75	14 (33%)	18 (46%)	NS
Plaque	14 (25%)	45 (53%)	< 0.05
ABI < 0.9	1 (2%)	11 (13%)	< 0.05

Data are shown as number (%)

*IMT* Intimo-Medial Thickness, *ABI* Ankle-Brachial Index, *NS* Not statistically significant

Table 2 shows the main characteristics of all patients, grouped according to whether or not they suffered from subclinical arteriosclerotic burden. Patients with ATB were older, were more often male, had a longer evolution of hypertension, were more likely to be using lipid-lowering drugs and suffered from a higher vascular risk. When analyzing the overall sample, a higher percentage of smokers and ex-smokers and a greater abdominal circumference are observed in patients with ATB, although these differences disappear when men and women are analyzed separately.

**Table 2:** Demographic and clinical features of patients

	All *n* = 140	ATB (yes) *n* = 91 (65)	ATB (no) *n* = 49 (35)	*p*
Age (years-old)	61,51 ± 9,2	62,6 ± 9,4	59,45 ± 8,57	0,05
Sex (men)	76 (54,3)	58 (63,7)	18 (36,7)	0,002
(women)	64 (45,7)	33 (46,3)	31 (63,3)	
Duration HTN (years)	7,64 ± 6,5	8,4 ± 6,5	6,08 ± 6,38	0,038
Smoking (present or past)	86 (61,4)	65 (71,4)	21 (42,9)	0,001
-Men	63 (73,3)	49 (75,4)	14 (66,7)	0,4
-Women	23 (26,7)	16 (24,6)	7 (33,3)	
Dyslipidemia on therapy				0,048
- No	94 (67,1)	56 (61,5)	38 (77,6)	
- Yes	46 (32,9)	35 (38,5)	11 (22,4)	
Risk category (Score)				0,002
- Moderate	57 (40,7)	28 (30,8)	28 (57,1)	
- High	83 (59,3)	63 (69,2)	21 (42,9)	
BMI (kg/mt^2^)	30 ± 4	30 ± 4	29 ± 5	NS
Abdominal circunference				
All	101,8 ± 10,7	103,8 ± 9,5	98,1 ± 11,9	0,003
Men	106,2 ± 8,6	106,2 ± 8,8	104,2 ± 7,7	0,2
Women	96,5 ± 10,7	98,4 ± 8,4	94,5 ± 12,6	0,1
LVH	13 (9%)	5 (10%)	8 (9%)	NS
Prediabetes		43 (47%)	21 (42%)	NS
Metabolic syndrome		34 (37%)	16 (32%)	NS
SBP (mmHg)	136 ± 15	136 ± 15	137 ± 13	NS
DBP (mmHg)	81 ± 8	81 ± 8	82 ± 9	NS

Data are shown as mean ± SD or *n* (%)

*ATB* Arteriosclerotic burden, *BMI* Body mass index, *HTN* Hypertension, *SBP* Systolic blood pressure, *DBP* diastolic blood pressure, *LVH* Left Ventricular Hypertrophy

Table 3 shows the analytical data, highlighting that ATB patients showed higher levels of creatinine and uric acid, whereas the rest of the parameters studied were similar in both groups, including frequency of APOE genotypes (rs429358 and rs7412) and LIPC (rs2070895). However, there was a non-significant higher prevalence of patients carrying the A allele (GA and AA) in patients with ATB than in those without it (44% vs 34%, NS).

**Table 3:** Analytical data

	TOTAL *n* = 140 (%)	ATB (yes)	ATB (no)	*p*
Glycemia (mg/dL)	92 ± 11,5	92,7 ± 10,9	90,2 ± 12,5	0,2
Creatinine (mg/dL)	0,84 ± 0,1	0,88 ± 0,18	0,77 ± 0,18	0,002
Uric acid(mg/dL)	5,2 ± 1,38	5,54 ± 1,4	4,77 ± 1,17	0,001
TSH (µUI/mL)	2,1 ± 1,3	2,1 ± 1,4	2,06 ± 1,2	0,66
Ferritin (ng/mL)	132 ± 107	129,8 ± 107	137,9 ± 109	0,67
hsCRP (mg/L)	3,3 ± 3,4	3,6 ± 3,5	2,6 ± 3,2	0,12
HbA1c (%)	5,6 ± 0,3	5,6 ± 0,3	5,5 ± 0,3	0,29
Cholesterol (mg/dL)	206 ± 34	204 ± 33,5	208 ± 36,7	0,58
HDL cholesterol (mg/dL)	52 ± 12	51 ± 11	55 ± 14	0,07
Women	57 ± 12	55 ± 10	59 ± 15	0,2
Men	48 ± 10	49 ± 10	48 ± 9	0,8
LDL cholesterol (mg/dL)	125 ± 33	124,8 ± 32	127,2 ± 35,4	0,6
< 55	2 (1.4)	0	2 (4.1)	
55–69	2 (1.4)	2 (2.2)	0	
70–99	25 (18)	16 (17)	9 (18)	
> 100	111 (79)	73 (80)	38 (78)	
Non HDL cholesterol (mg/dL)	153 ± 35	153,6 ± 35	153 ± 39	0,9
Triglycerides (mg/dL)	140 ± 66	145,8 ± 71	131 ± 57	0,22
Apolipoprotein A1 (mg/dL)	140 ± 10	140 ± 9,4	142 ± 11	0,22
Apolipoprotein B (mg/dL)	102 ± 15	103 ± 14	102 ± 17	0,73
Apolipoprotein B48 (mg/dL)	9,8 ± 4,7	9,5 ± 4,7	10,4 ± 5	0,27
*Apo E* Genotype*				0,2
- 2/2	1 (0,7)	9 (9,9)	1 (2,0)	
- 2/3	12 (8,6)	67 (73,6)	3 (6,1)	
- 3/3	100 (71,4)	13 (14,3)	33 (67,3)	
- 3/4	24 (17,1)	2 (2,2)	11 (22,4)	
- 4/4	2 (1,4)			
- 2/4	1 (0,7)		1 (2,0)	
*LIPC* Genotype*				0,3
(GG)	83 (59,3)	51 (56)	32 (65,3)	
(GA)	50 (35,7)	34 (37,4)	16 (32,7)	
(AA)	7 (5)	6 (6,6)	1 (2,0)	

Data are shown as mean ± SD or n(%)

Table 4 shows the multivariate analysis, where the variables that were significantly associated with ATB were smoking or having smoked, creatinine levels and the evolution time of arterial hypertension. In contrast, the age and smoking variables were associated with carotid plaque (Table 5).

**Table 4:** Binary lineal regression analyses taking arteriosclerosis burden as dependent variable

Factor	OR (95% confidence interval)
Smoking (past or present vs never)	3.68 (1.65–8.21)
Serum creatinine (by mg/L)	1.33 (1.06–1.66)
Hypertension duration (by year)	1.10 (1.02–1.18)

Variables not included in the equation: age, sex, prediabetes status, metabolic syndrome, neutrophils, platelets, glycemia, uric acid, non-HDL cholesterol, HDL cholesterol, triglycerides, apolipoprotein B, apolipoprotein B48, microalbuminuria, hsCRP, *LIPC* genotype (AA plus GA vs GG) and apolipoprotein E genotype (E3/3 vs no E3/3)

**Table 5:** Binary linear regression analyses taking carotid plaque as dependent variable

Factor	OR (95% confidence interval)
Smoking (past or present vs never)	3.805 (1.67–8.65)
Age (by year)	1.088 (1.041–1.136)

Variables not included in the equation: duration of hypertension, creatinine, uric acid, sex, prediabetes status, metabolic syndrome, neutrophils, platelets, glycemia, uric acid, non-HDL cholesterol, HDL cholesterol, triglycerides, apolipoprotein B, apolipoprotein B48, microalbuminuria, hsCRP, *LIPC* genotype (AA plus GA vs GG) and apolipoprotein E genotype (E3/3 vs no E3/3)

Table 6 presents the distribution of cholesterol levels in the sample segregated by the re-classified VC risk level, showing that 3 out of 4 patients had an LDL-C level > 100 mg/dL unrelated to their risk level. In general terms, only 1 out of 3 or 4 hypertensive patients was undergoing lipid-lowering treatment, always with statins in monotherapy. There was only 1 patient being treated with the statin and ezetimibe combination therapy. Patients receiving statin treatment (*n* = 42) had lower LDL-C (114 ± 32 mg/dL) than those who were not taking statins (131 ± 33 mg/dL, *p* < 0.05). In contrast, most patients were taking one or two drugs to control their BP, with more than 50% of patients reaching an optimal control (Table 6).

**Table 6:** LDL cholesterol levels and blood pressure control according to new CV risk category, after testing for subclinical arteriosclerosis

	CARDIOVASCULAR RISK CATEGORY			
	**MODERATE**	HIGH	VERY HIGH	*p*
LDLc (mg/dL)	122 ± 33	133 ± 33	123 ± 33	NS
< 55	2 (5)	0	0	
55–69	0	0	2 (3.4)	
70–99	7 (17)	6 (15)	12 (20)	
> 100	31 (77)	35 (85)	45 (76)	
LLT				NS
None	29 (72)	32 (78)	34 (58)	
Statins	9 (22)	9 (22)	24 (41)	
BP control	23 (58)	20 (51)	36 (61)	NS
Drugs for BP				NS
Life-style	5 (12)	5 (12)	5 (8)	
1	17 (43)	18 (44)	22 (37)	
2	14 (29)	14 (29)	20 (41)	
3	4 (10)	3 (7)	10 (17)	
4	0	1 (2)	2 (3)	

Data are shown as number (%) or mean ± SD

*LLT* Lipid-lowering therapy, *BP* Blood pressure controlled at 6 months, *NS* Not statistically significant

## Discussion

This study confirms that in hypertensive non-diabetic patients in primary prevention with a moderate or high VR, looking for subclinical vascular disease using conventional ultrasound, a technique available in primary care centers, has allowed us to identify 91 patients (65%) with atherosclerotic subclinical burden in a sample of 140 patients. Patients with ATB have an increased risk of premature mortality not only in the elderly [5] but also in middle-aged adults [6]. The results of previous studies showed that all three components (IMT, carotid plaque and left ventricular hypertrophy) of ATB contributed similarly and significantly to premature mortality, suggesting that premature mortality is not driven solely by just one or two components [5, 6].

The prevalence of carotid plaques (41%) was high although similar to that shown in other research studies carried out in Spain [35]. It is worth stressing that 25% of patients with moderate VR had plaques, which is also consistent with other published studies [4].

Identifying patients with carotid plaque changed the level of risk; consequently 59 patients (42%) were re-classified as having a very high VR, and were hence eligible for intensified preventive measures [2]. Clinical practice guidelines in Europe establish that re-classification is of value in people identified as being at moderate VR by using markers such as CAC score > 100 Agatston units, ankle-brachial index (ABI) < 0.9 or > 1.40, carotid/femoral pulse wave velocity > 10 m/s, or the presence of plaques at carotid or femoral ultrasonography [2]. Out of 12 patients with ABI < 0.9, 11 patients had carotid plaque and the remaining one showed a high IMT.

Taking our data into account, in hypertensive non-diabetic patients, the better technique to re-classify patients is carotid ultrasound rather than ABI measurement, as the former detects subclinical atherosclerosis in earlier stages. In the literature there are data that recommend the routine performance of ABI measurement preferably in patients who have symptoms of intermittent claudication, as well as asymptomatic subjects who are diabetic, smoke, and are over 50 (ADA 2021). The European Society of Vascular Surgery recommends ABI measurement in asymptomatic individuals who are clinically-free but at high risk of lower extremity arterial disease, in the following cases: men and women aged > 65, those below 65 classified at high cardiovascular risk according to the ESC guidelines, and men and women aged > 50 with a family history of peripheral arterial disease [36].

The multivariate analyses showed that, in addition to smoking (a well-known fact in the literature) [37, 38], the factors independently associated with ATB were different (i.e. serum creatinine, duration of hypertension) from those associated with the presence of carotid plaque (i.e. age). The main difference is that ATB, as defined in this study, includes, in addition to plaques, an IMT > 75% adjusted to age and sex. There is considerable debate about the value of measuring carotid IMT, as some practice guidelines recommend not using it to re-classify patients from moderate to high or very high risk [2, 39]. Some even argue that a high IMT value should not be considered as subclinical arteriosclerosis but rather as an effect of the thickening of the arterial wall as a result of aging [40, 41]. Our study results suggest a greater relationship between the hemodynamic effect of hypertension (duration of the disease and renal dysfunction) and a high IMT value than with the former and the presence of plaques. Furthermore, the fact that both IMT and plaque largely share the same risk factors suggests that they are really two phases of the same atherosclerotic process, as pointed out by some studies [35, 42, 43].

Contrary to what was initially expected by our research team [21, 31, 44], we did not find any relationship between the levels of subclinical arteriosclerosis burden, IMT or carotid plaque and the levels of Apo B-48. Fasting levels of Apo B-48 are frequently associated with high triglyceride levels and type 2 diabetes, where an increase in the intestinal production of Apo B-48 in the fasting state is described. This study has shown triglyceride levels mostly below 200 mg/dL and had excluded diabetes mellitus from the inclusion criteria.

Genetic predisposition plays a major role in the development of atherosclerosis, not only related to cardiovascular risk factors, such as lipid levels or blood pressure [45], but also associated with the arterial wall such as endothelial damage and dysfunction, an early component of atherosclerosis [46]. In our study, the multivariate analysis did not find any relationship between the LIPC gene variants and the subclinical arteriosclerotic disease. However, a univariate analysis did find that 44% of patients with subclinical arteriosclerosis show at least one A allele in the *LIPC* gene, as compared to only 34% of patients who had no burden. This may be due to the fact that our research was not specifically designed to address the study of this variable. However, despite of recognizing the role of genetic variation in the pathogenesis of atherosclerosis, the polygenic nature of individuals as well as the multifactorial causality of atherosclerosis it is very difficult in a limited group of subjects that genetic analyses might be integrated to conventional risk stratification models. As an example, the polygenic score (based in 12 SNPs) in severe hypercholesterolemia subjects did not improve predictive accuracy when added to the readily available characteristics age, sex and LDL-C, suggesting limited discriminative value for CAD [47].

Regarding treatment, it is worth highlighting that 6 out of 10 patients had good blood pressure control, probably because many were taking two or more hypotensive drugs, despite of many patients consider hypertension as a risk factor and no as a disease [48]. The combination of antihypertensive drugs with different mechanisms of action has shown great efficacy in the treatment of hypertensive patients. In contrast, our study shows a clear undertreatment in the prescription of lipid-lowering drugs. Less than 30% of the patients were being treated with statin monotherapy at the time of the study, and only 1 patient was being treated with statin + ezetimibe. These results are in line with other studies carried out in primary prevention in Europe [49], where monotherapy is most common. In order to achieve the LDL-C levels that are currently recommended by European bodies [2], and considering the high VR of patients before and, especially, after being re-classified, it is clear that many patients should be treated with a statin-ezetimibe combination, in order to find optimal lipid treatment [50]. In fact, 55% of patients treated with this combination in primary prevention achieved LDL-C < 70 mg/dL, according to the Da Vinci research study. Several studies, using statin plus ezetimibe [51] or statin plus PCSK9i [52, 53] have shown clinical benefits in terms of preventing coronary and cerebrovascular events when compared with statin in monotherapy”. Because PCSK9i combination in Spain is restricted to patients in secondary prevention or with familial hypercholesterolemia, and is therefore not accessible in primary prevention, the drug of choice should be ezetimibe added to statins.

From a clinical point of view, the presence of subclinical atherosclerosis in a hypertensive patient should improve the quality of prescription to control not only blood pressure but also hypercholesterolemia, via reducing therapeutic inertia, and to motivate patients to improve adherence and compliance with diet, life-style and drug recommendations.

This research study has several limitations. Firstly, this was not a population-based study with a random sample, as patients were not randomly selected. Instead, the sample consisted of patients who attended medical appointments, which could affect the external validity of the results. It is a cross-sectional study, which prevents establishing causal relationships. Secondly, the characteristics of the plaques have not been considered in the study. Assessment of plaque echogenicity improves CVD risk prediction. Thus, the VR (coronary ischemic disease, ischemic stroke and peripheral arterial disease) is higher when echolucent carotid plaques are detected [37]. Third, the study did not assess the pulse wave velocity, which is a strong predictor of vascular events [54, 55]; the test was not available at the moment of the study was carried out. A strength of this study is that the use of automated software has made it possible to obtain very accurate measurements, unlike other previous studies.

## Conclusions

Non-invasive diagnostic techniques, such as carotid ultrasound, are very effective in detecting subclinical damage that leads to re-classifying VR in a great number of hypertensive patients.

## Data Availability

The data that support the findings of this study are available from the corresponding author, PV, upon reasonable request.
